# Incongruent pitch cues are associated with increased activation and functional connectivity in the frontal areas

**DOI:** 10.1038/s41598-018-23287-5

**Published:** 2018-03-26

**Authors:** Jo-Fu Lotus Lin, Toshiaki Imada, Patricia K. Kuhl, Fa-Hsuan Lin

**Affiliations:** 1 Institute of Biomedical Engineering, National Taiwan University, Taipei, Taiwan; 2 Institute for Learning & Brain Sciences, University of Washington, Seattle, WA USA; 3 Department of Speech & Hearing Sciences, University of Washington, Seattle, WA USA

## Abstract

Pitch plays a crucial role in music and speech perception. Pitch perception is characterized by multiple perceptual dimensions, such as pitch height and chroma. Information provided by auditory signals that are related to these perceptual dimensions can be either congruent or incongruent. To create conflicting cues for pitch perception, we modified Shepard tones by varying the pitch height and pitch chroma dimensions in either the same or opposite directions. Our behavioral data showed that most listeners judged pitch changes based on pitch chroma, instead of pitch height, when incongruent information was provided. The reliance on pitch chroma resulted in a stable percept of upward or downward pitch shift, rather than alternating between two different percepts. Across the incongruent and congruent conditions, consistent activation was found in the bilateral superior temporal and inferior frontal areas. In addition, significantly stronger activation was observed in the inferior frontal areas during the incongruent compared to congruent conditions. Enhanced functional connectivity was found between the left temporal and bilateral frontal areas in the incongruent than congruent conditions. Increased intra-hemispheric and inter-hemispheric connectivity was also observed in the frontal areas. Our results suggest the involvement of the frontal lobe in top-down and bottom-up processes to generate a stable percept of pitch change with conflicting perceptual cues.

## Introduction

Most auditory stimuli in our everyday experience are natural and complex sounds with multiple acoustic features. To understand complex auditory stimuli, such as music and speech, pitch perception plays a crucial role. Pitch perception in music is associated with multiple dimensions, including pitch height and pitch chroma^1–3^. Pitch height describes a linear dimension related to the fundamental frequency of a sound. Pitch chroma describes a cyclic dimension related to the relative position within the frequency range of an octave^2,4,5^.

Studies have varied different perceptual dimensions of pitch to investigate their relative importance in music and speech perception^6–10^. More specifically, pitch cues related to these perceptual dimensions can be designed to provide congruent or incongruent information. For example, missing fundamental complexes^11,12^ were composed of harmonics that were multiples of the lowest frequency component, but not the fundamental frequency. When the spectral pitch was shifted up and the missing fundamental was shifted down, a situation of conflicting perceptual cues was created^7^. Two listening modes have been reported^13,14^. Listeners in the analytic or spectral listening mode responded to individual harmonics or spectral pitch, whereas those in the holistic or synthetic listening mode reconstructed and inferred the missing fundamental frequency^15–17^.

One intriguing example of varying pitch height and pitch chroma is the Shepard tones which lead to the perception of endlessly ascending or descending pitch^18^. Pitch perception of Shepard tones was first systematically investigated by Shepard^18^ using complex harmonic tones composed of harmonic partials separated by one octave. The amplitudes of these harmonic partials were scaled by a bell-shaped envelope. The spectral shape and position remained constant across complex tones. The perception of continuously rising or falling pitch was elicited by simply repeating a sequence of such Shepard tones. Shepard tones have been modified and incorporated to create sound effects in popular music, video games, and movies^19^.

**Figure 1:**
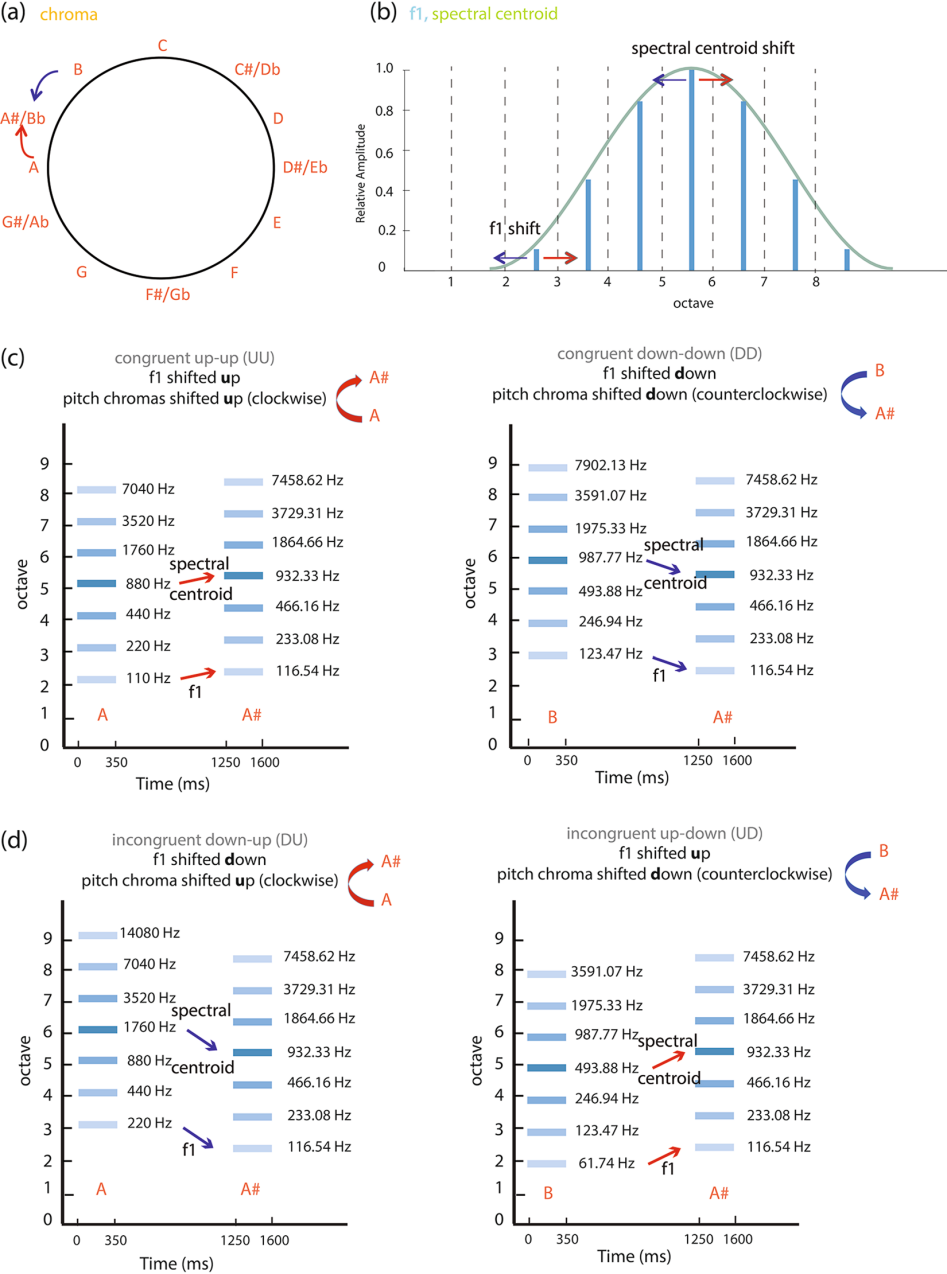
Schematic illustrations of the complex harmonic tones. **(a**) A change in pitch chroma in clockwise direction along the chroma circle is perceived as an upward pitch shift (arrows in red), whereas a change in counterclockwise direction is perceived as a downward pitch shift (arrows in blue). **(b)** Spectral envelopes and harmonic partials along the octave scale are plotted. Vertical lines represent octave-spaced harmonic partials, all of which share the same chroma as the center of the spectral envelope. A shift in the lowest harmonic or the harmonic partials is perceived as a shift in pitch height. In our study, the pitch shift direction in harmonic partials is always the same as that in spectral envelope. Amplitudes of harmonic partials are weighted by a Gaussian envelope. **(c)** Two complex tones are played sequentially and their harmonic partials are shown schematically. Lighter colors denote smaller relative amplitudes compared to the center harmonic partial. In the congruent conditions, the direction of change in pitch height matches that in pitch chroma. **(d)** In the incongruent conditions, the direction of change in pitch height is in a conflicting direction with pitch chroma.

Pitch perception of Shepard tones has been related to the Gestalt principle of proximity^20,21^. Specifically, when presented with Shepard tones, listeners tend to make pitch judgments based on pitch chroma or chroma proximity, which is described by the closeness in the pitch space of a helical model^2^. That is, the perceived direction of pitch change depends on the shortest log–frequency distance between sounds^18^. For example, by visualizing 12 pitch classes in a chroma circle with C at 12 o’clock, D# at 3 o’clock, F# at 6 o’clock, and A at 9 o’clock (Fig. 1a), the distance between A and A# is one semitone in the clockwise or upward direction, but 11 semitones in the counterclockwise or downwards direction. Listeners relying on chroma for pitch perception tend to report an upward pitch in this case.

When judging the relative pitch of a pair of sounds, listeners usually assign different weights to perceptual cues or dimensions^1,15,22^. When cues from one auditory perceptual dimension are weak or ambiguous, listeners assign larger weights to other dimensions to make pitch judgments. The resulting percept may remain stable or alternate between two mutually exclusive interpretations over time^23^. In Shepard tones, complex sounds are composed of varying harmonic partials with a fixed spectral envelope^18^. Because these complex harmonic tones are octave-spaced and share the same chroma, pitch chroma is well-defined. Previous studies have argued that these octave-space harmonics result in ill-defined or ambiguous pitch height because harmonic partials belong to different octave positions^18,19^. Thus, for the typical Shepard tones, the importance of pitch height in pitch judgments is reduced and pitch chroma becomes a more prominent perceptual cue. We speculate that pitch perception of Shepard tones can be explained by listeners relying on pitch chroma more than on pitch height to generate a percept of pitch.

A few neuroimaging studies have investigated neural correlates underlying auditory pitch perception caused by Shepard tones. With functional magnetic resonance imaging (fMRI), Shimizu *et al*.^24^ reported that not only the auditory cortex but also occipital regions showed significant hemodynamic responses to a sequence of ascending Shepard tones. In an event-related potential (ERP) study, Tervaniemi *et al*.^25^ used a sequence of descending Shepard tones in an auditory mismatch negativity design. Their results showed that abstract stimulus features, such as the descending pattern, were encoded in the neural representation of sensory memory. In both studies, a sequence of Shepard tones was played to facilitate building up a context of an ascending or descending sequence of sounds. In an ascending sequence of Shepard tones when we move from one complex harmonic to another, such as from complex harmonic A to A#, all harmonic partials are shifted up along the same direction as pitch chroma. In this case, congruent cues of pitch height and pitch chroma are provided. However, when the sequence goes from the last tone back to the first tone of a Shepard tone sequence, for example from complex harmonic B to C, all harmonic partials are shifted down but pitch chroma is shifted up. Incongruent perceptual cues of pitch height and pitch chroma are provided in such a case. Therefore, the cortical activation observed when a subject is listening to a sequence of Shepard tones can be related to the processing of both incongruent and congruent cues. Moreover, in both studies, no direct comparisons were made between perceiving pitch with congruent and incongruent cues. No behavioral responses were collected to ensure that pitch with incongruent cues was resolved and whether a stable percept was created.

The goal of our present study was to examine pitch perception in terms of (*i*) whether listeners weight pitch height or pitch chroma cues more strongly to make pitch judgments when two cues provide conflicting information, and (*ii*) what cortical areas are associated with solving conflicting perceptual cues in pitch compared to non-conflicting situations.

The original Shepard tones had varied harmonic partials but a fixed spectral envelope, providing a more salient pitch chroma cue than pitch height cue. In our experiment, we modified Shepard tones by varying both the harmonic partials and spectral envelopes in the same direction in frequency. That is, when harmonic partials were shifted up, the spectral centroid or envelope was also shifted up. In addition, we varied pitch height and pitch chroma in the same or conflicting directions, resulting in congruent or incongruent perceptual cues of pitch shift, respectively. In the congruent conditions, the pitch shift direction in pitch height (e.g., upwards) matched that in pitch chroma (e.g., upwards). In the incongruent conditions, the shift in pitch height (e.g., downwards) conflicted with that in pitch chroma (e.g., upwards). We hypothesized that when the perceived directions of change in pitch height and pitch chroma are consistent (upwards or downwards), a clear percept of pitch change (upward or downward) would emerge. On the other hand, when conflicting cues in pitch height and pitch chroma are provided, percepts of pitch shift would then depend on the relative weights listeners assign to the pitch height and pitch chroma dimensions. By using a pitch judgment task in which listeners were asked to judge the pitch direction of a pair of our modified Shepard tones, we can infer whether listeners weight the pitch height or pitch chroma cues more strongly based on their behavioral responses. For example, given a sound pair with pitch height shifted upwards but pitch chroma shifted downwards, weighting the pitch height dimension more strongly would lead to perceiving an upward pitch shift. Weighting the pitch chroma dimension more strongly would lead to a percept of downward pitch change. Reliance on pitch height suggests the analytic listening mode, wherein listeners pay attention to fine structures in the acoustic stimuli. Reliance on pitch chroma suggests the holistic listening mode, wherein one percept for the entire complex harmonic tone is generated.

In contrast with previous studies using a block design fMRI or a mismatch design in ERP^24,25^, our experimental design created conflicting cues simply by using a pair of complex tones, instead of playing a sequence of Shepard tones. With paired tones, we shortened the trial duration and were allowed to collect trials with congruent and incongruent cues of pitch in the same testing session. For pitch height and pitch chroma processing, previous fMRI studies have reported either distinct or overlapped activation in the planum temporale (posterior to Heschl’s gyrus) and planum polare (anterior part of the temporal lobe)^5,26^. Pitch perception also involves cognitive processes, beyond perceptual mechanisms^27^, especially when resolving conflicting perceptual cues. By contrasting incongruent versus congruent conditions, we aimed to study whether auditory cortex or higher-level processing areas are activated to solve conflicting pitch cues.

## Results

Subjects were asked to make pitch judgments based on two complex harmonic tones presented sequentially. We measured behavioral responses and fMRI blood-oxygen-level-dependent (BOLD) signals simultaneously. Our experiment included four conditions with different combinations of changes in pitch chroma and pitch height (Fig. 1; see Methods for details). These four conditions included ***(1) congruent condition up-up (UU)***: a pair of sounds with an upward change in pitch height and pitch chroma, ***(2) congruent condition down-down (DD)***: a pair of sounds with a downward shift in pitch height and pitch chroma, ***(3) incongruent condition down-up (DU)***: a pair of sounds with a downward shift in pitch height but an upward shift in pitch chroma, and ***(4) incongruent condition up-down (UD)***: a pair of sounds with an upward movement in pitch height but a downward movement in pitch chroma.

### Behavioral Results

To ensure that subjects accurately judged changes in pitch instead of guessing, subjects with less than 90% accuracy in the congruent conditions (UU and DD) were excluded from further analysis. Five of 21 subjects were thus excluded. From the remaining 16 subjects, the mean accuracy of pitch judgments was 97.1% +/− 3.5% (mean +/− SD), ranging from 90.7% to 100% in the congruent conditions when the direction of change in pitch height matched that in pitch chroma. For fMRI analysis, trials with incorrect pitch judgments were removed. The discarded trials were those during which listeners reported a downward pitch shift when both the pitch height and chroma were shifted upwards, or reported an upward pitch movement when both the pitch height and chroma were shifted downwards.

**Figure 2:**
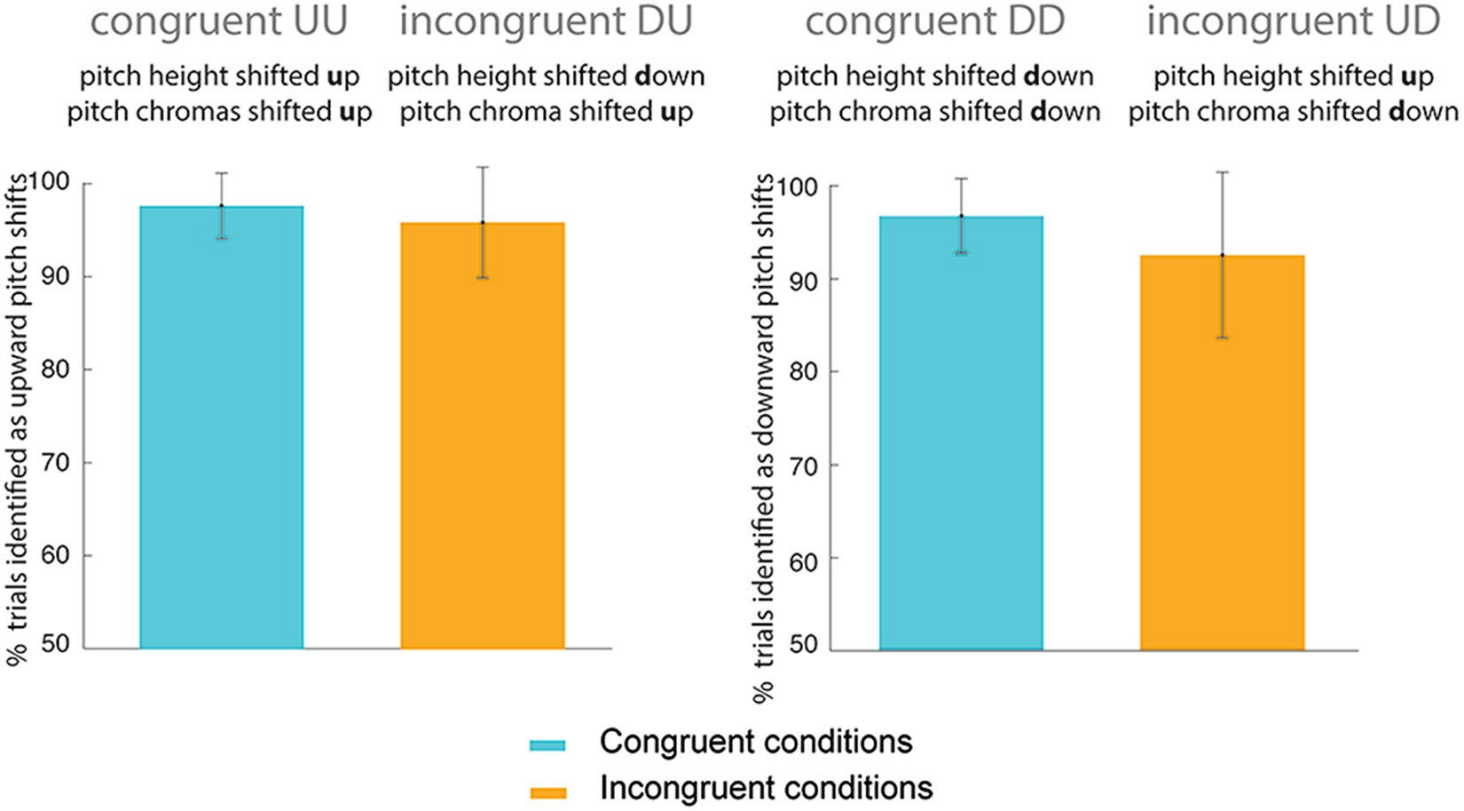
Behavioral results of the congruent and incongruent trials. Bar heights denote the mean percentage of trials in which an upward or downward pitch shift was perceived in the congruent (UU, DD) and incongruent (DU, UD) conditions. Error bars represent one standard deviation.

Figure 2 shows the percentage of trails in which an upward or downward pitch movement was perceived in the congruent (UU, DD) and incongruent (DU, UD) conditions. No significant difference was found between the congruent (97.1% +/− 3.5%) and incongruent conditions (94.2% +/− 6.9%) (p = 0.078).

In the incongruent conditions when complex tone pairs were presented with a downward shift in pitch height but an upward shift in pitch chroma (incongruent DU condition), listeners perceived an upward pitch shift in 95.8% +/− 6.0% of the trials. Only a small portion of the trials (4.2%) was associated with a percept of downward pitch shift. When complex tone pairs with an upward shift in pitch height but a downward shift in pitch chroma were presented (incongruent UD condition), listeners perceived a downward pitch movement in 92.4% +/− 8.9% of the trials. Listeners perceived an upward pitch movement in 7.6% of the trials. Averaging across incongruent conditions (DU and UD), pitch judgments depended on the pitch chroma cue in 94.2% +/− 6.9% of the trials, ranging from 75.9% to 100%. That is, listeners showed consistent responses based on the pitch chroma cue, rather than on the pitch height cue. This result suggested that listeners had stable pitch percepts across trials during the incongruent conditions, instead of switching between two percepts or relying on different perceptual cues across trials. Thus, for fMRI analysis, we only included those trials with stable percepts to ensure that the same perceptual cue (pitch chroma) was used. This criterion resulted in at least 42 valid trials included per condition. Percepts based on the pitch height cue were not analyzed because there were less than 25% of the trials, which was less than 10 trials.

### fMRI Results

#### Task versus baseline

**Figure 3:**
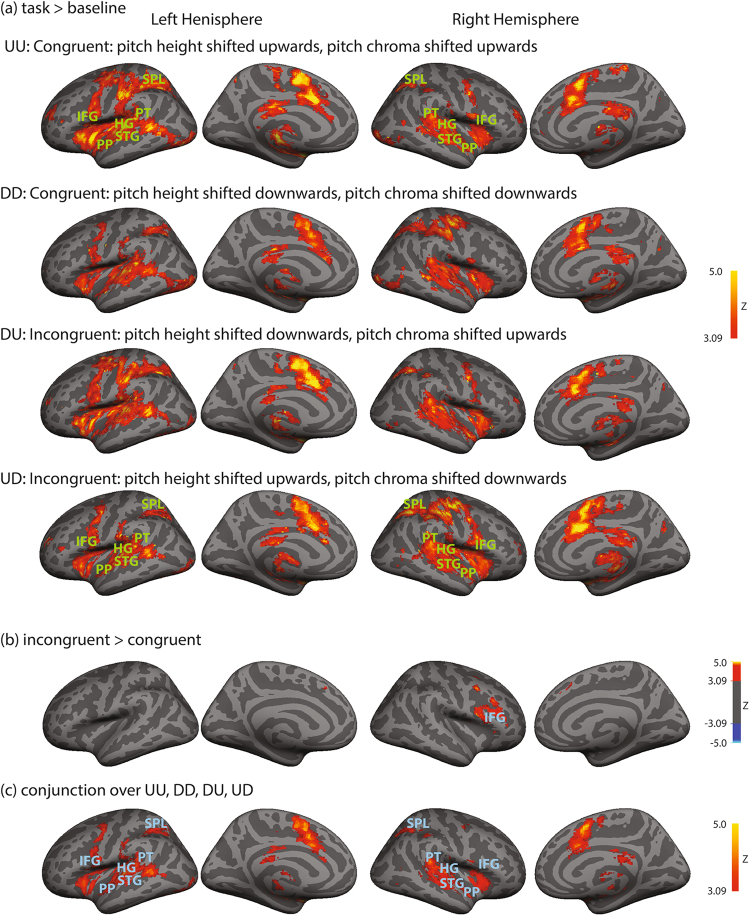
fMRI results. Group statistical maps are displayed on inflated cortical surfaces. Group-level activation maps of (**a**) task (UU, DD, DU, or UD) versus baseline, (**b**) significant differences between the incongruent and congruent conditions, and (**c**) conjunction analysis among four conditions (UU, DD, DU, UD). IFG: inferior frontal gyrus, SPL: superior parietal lobule, PP: planum polare, PT: planum temoprale, HG: Heschl’s gyrus, STG: superior temporal gyrus.

To find brain regions sensitive to pitch changes, activation during task conditions were compared to baseline. Four contrasts were tested, including UU versus baseline, DD versus baseline, DU versus baseline, and UD versus baseline. All contrasts revealed activation in the bilateral temporal lobe (planum polare, planum temporale, Heschl’s gyrus, superior temporal gyrus, middle temporal gyrus), frontal lobe (inferior frontal gyrus, middle frontal gyrus, precentral gyrus), parietal lobe (superior parietal lobule, supramarginal gyrus), and cingulate gyrus (Fig. 3a).

#### Incongruent versus congruent conditions

To probe cortical areas associated with solving incongruent pitch cues, we contrasted BOLD signals between incongruent (DU, UD) and congruent conditions (UU, DD). We found the following areas were significantly more activated during the incongruent compared to congruent conditions: the right inferior frontal gyrus (pars opercularis, pars triangularis), middle frontal gyrus, and the cingulate gyrus (Fig. 3b; Table 1). No areas were significantly more activated during the non-incongruent than congruent conditions of pitch change.

**Table 1:** Brain regions showing significant differences in activation between the incongruent and congruent conditions. Peak activation of significant clusters from the contrast of incongruent > congruent conditions are listed. Coordinates in the MNI space, number of voxels in a cluster, and maximum z-values are listed.

Incongruent > Congruent	Cluster extent (voxels)	max Z-score	peak MNI coordinates		
Region			x	y	z
Right inferior frontal gyrus (opercularis)	1146	4.75	50	10	14
Paracingulate gyrus	338	4.19	6	28	40

#### Common activation across conditions

To identify regions commonly activated among the congruent and incongruent conditions, we used conjunction analysis for the four tasks versus baseline contrasts (UU versus baseline, DD versus baseline, DU versus baseline, UD versus baseline). Significantly common activation was observed bilaterally in the inferior frontal gyrus (pars opercularis), superior temporal gyrus, planum polare, planum temporale, Heschl’s gyrus, superior parietal lobule, precentral gyrus, and anterior cingulate gyrus (Fig. 3c; Table 2).

**Table 2:** Brain regions showing significant conjunction effect for the congruent and incongruent conditions.

Conjunction analysis	Cluster extent (voxels)	max Z-score	peak MNI coordinates		
Region			x	y	z
Left superior temporal gyrus, middle temporal gyrus, parietal, frontal cortices, cingulate gyrus	20476	5.69	−22	4	−4
Left inferior temporal, middle temporal gyrus	1379	5.09	−35	−66	−21
Right inferior temporal gyrus	902	5.47	36	−58	−26
Right cingulate gyrus	567	4.09	6	−30	24
Right superior parietal lobule	431	4.31	30	−52	38
Right precentral gyrus, middle frontal gyrus	74	3.75	36	−2	50
Right inferior frontal gyrus, precentral gyrus	47	3.87	54	8	12

#### Psychophysiological interaction analysis

**Figure 4:**
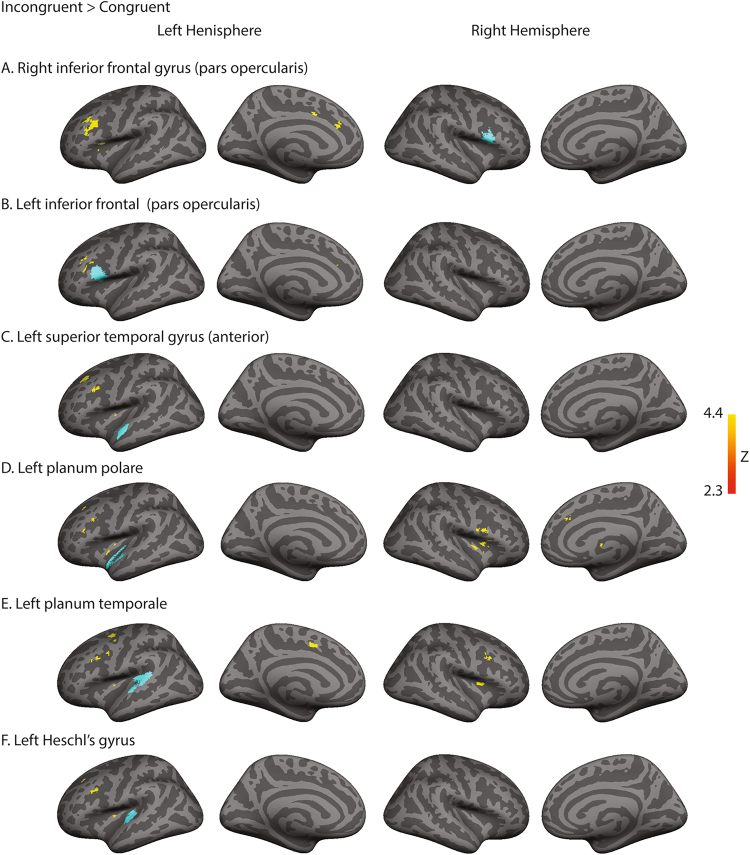
Results from the psychophysiological interaction (PPI) analysis. Light blue color shows anatomically-defined seed regions. Seed regions included the left and right inferior frontal gyrus (pars opercularis), superior temporal gyrus (anterior), planum porale, planum temporale, and Heschl’s gyrus. Red/yellow colors indicate stronger connectivity with the seed regions during the incongruent than congruent conditions.

To further reveal the bottom-up and top-down information flows between cortical areas, we used psychophysiological interaction (PPI) analysis to find differences in functional connectivity between the congruent and incongruent conditions. Regions showing common activation across the four conditions from the conjunction analysis were used as seed regions, including the inferior frontal gyrus (pars opercularis), superior temporal gyrus (anterior), planum porale, planum temporale, and Heschl’s gyrus in both hemispheres. Significantly stronger connectivity was observed during the incongruent compared to the congruent conditions. Specifically, the left temporal seed regions (planum temporale and planum polare) exhibited enhanced connectivity with the frontal regions bilaterally. Increased connectivity was also observed between the bilateral frontal and the left middle frontal gyrus (Fig. 4; Table 3). No significantly stronger connectivity was found with the seed regions for the congruent compared to incongruent trials.

**Table 3:** Brain region showing significantly enhanced connectivity with seed regions (A to F) in the incongruent compared to the congruent conditions.

Incongruent > Congruent	Cluster extent (voxels)	max Z-score	peak MNI coordinates		
Area			x	y	z
**A. Right inferior frontal gyrus (opercularis)**					
Left middle frontal gyrus, cingulate gyrus	1063	3.62	−26	18	20
**B. Left inferior frontal gyrus (opercularis)**					
Left middle frontal gyrus	270	3.3	−27	22	24
**C. Left superior temporal gyrus (anterior)**					
Left middle frontal gyrus	521	3.35	−26	17	34
Insula	353	3.22	−24	−3	3
**D. Left planum polare**					
Right insula, inferior frontal gyrus (opercularis)	1063	3.55	26	6	10
Left insula, inferior frontal gyrus (opercularis)	485	3.44	−26	20	32
Left middle frontal gyrus	414	3.14	−25	18	30
**E. Left planum temporale**					
Right inferior frontal gyrus (opercularis)	631	3.31	32	10	14
Left middle frontal gyrus, inferior frontal gyrus (opercularis)	596	3.21	−28	16	34
Left insula	367	3.45	−26	−8	6
**F. Left Heschl’s gyrus**					
Insula	361	3.36	−25	2	3
Left middle frontal gyrus, inferior frontal gyrus	310	3.34	−30	16	32

## Discussion

In our study, we varied pitch chroma and pitch height in conflicting directions to create conflicting cues of pitch shift. Compared to the typical Shepard tones, our complex tones were generated by varying harmonic partials and shifting spectral envelopes and the centroid in the same direction. In addition, pitch chroma was varied independently of pitch height. The pitch chroma cue was shifted either in the same direction as pitch height for the congruent conditions, or in conflicting directions for the incongruent conditions. In summary, our behavioral results from the incongruent conditions (Fig. 2) suggest a reliance on the pitch chroma cue, rather than pitch height cue, during pitch judgment tasks when pitch height and chroma provided conflicting cues. Comparing fMRI activation maps during the incongruent versus congruent conditions (Fig. 3), we found significantly stronger cortical activation in the right inferior frontal areas. Enhanced connectivity was observed from the left temporal areas to bilateral frontal areas in the incongruent than congruent conditions. Stronger intra-hemispheric and inter-hemispheric connectivity was also observed in the frontal areas (Fig. 4). Results from our present study reveal cortical areas that may be involved in resolving conflicting perceptual cues and creating a stable percept of pitch shift.

Based on stimulus characteristics of our complex tone pairs, we categorized trials into congruent (when the pitch shift direction in pitch height matched that in pitch chroma) and incongruent conditions (when pitch height was shifted in a conflicting direction with pitch chroma). We found that listeners tended to report one pitch shift direction in the incongruent conditions when contradicting cues were presented. Specifically, in the incongruent condition when pitch chroma shifted upwards but pitch height shifted downwards, listeners perceived an upward pitch shift. In the incongruent condition when pitch chroma shifted downwards but pitch height shifted upwards, listeners perceived a downward pitch shift. Thus, our behavioral results indicate that pitch chroma plays an important role in determining pitch change directions. The reliance on pitch chroma results in a stable percept of pitch movement across trials and subjects, rather than shifting between percepts across trials. Pitch chroma reflects the relative distance in the perceptual space. Based on the helix model proposed in music perception^1,2,28^, listeners tend to rely more on chroma differences within one octave, rather than absolute differences in frequency in the physical domain. In our study, complex tones were composed of octave-spaced harmonic partials with the same pitch class. Therefore, pitch class or chroma was clearly defined. Our results were consistent with previous studies of octave ambiguity in which sounds of the same chroma in different octaves were found to be musically similar^21,29^. The perceptual similarity or equivalence of sounds with the same chroma but varying octaves underlies the mechanism we use to identify melodies transposed into different octaves or played by instruments of different timbres^30,31^. The reliance on pitch chroma in contrast to pitch height is in line with findings that pitch chroma, or relative information in pitch, facilitates melody recognition and music perception^32,33^.

Our finding that most of our subjects relied on the pitch chroma cue resembles the holistic listening mode of missing fundamentals^13,15^. When listening to our complex harmonic tones, listeners fused all harmonic partials into one perceptual entity as one pitch class or chroma. Interestingly, several studies have indicated that linguistic backgrounds and music experience affect pitch perception^15,34,35^. For missing fundamentals, listeners of tonal languages tend to perceive pitch changes based on fundamental pitch^36^ or the holistic listening mode. Schneider *et al*.^17^ also reported that the listening mode depended on instruments or types of music musicians were trained with. Our results suggest the possibility that the reliance on pitch chroma is related to the language background of listeners. Note that the native language of our listeners is Mandarin Chinese, which is a tonal language.

Compared with previous studies using passive listening tasks^24,25^, the pitch judgment tasks used in our study required active responses. With behavioral responses, we analyzed brain activation during trials when a stable percept was elicited in the incongruent conditions. By directly comparing incongruent versus congruent conditions of pitch change, common as well as differential brain hemodynamic responses were observed (Fig. 3). Specifically, common activation was found in the bilateral superior temporal and inferior frontal areas. Given the pitch judgment tasks used in our study, sensory processing of auditory stimuli was required in both the incongruent and congruent conditions. The common brain activity found in the auditory areas may thus reflect bottom-up sensory processing of auditory features and early perceptual analysis of pitch^37^.

Common activation associated with pitch processing was also found in the inferior frontal areas, beyond the primary auditory cortex. The inferior frontal activation may be associated with processes required for pitch judgments, such as auditory working memory. In the previous fMRI study using Shepard tones^24^, activation in the bilateral primary auditory areas, not frontal regions, was reported. However, no behavioral responses were obtained from subjects in this previous study. Compared with previous studies, common activation found in the inferior frontal areas in our study can be associated with active pitch judgments during attended tasks when subjective responses are required. The frontal activation has been reported in PET and fMRI studies with pitch judgment tasks^38–40^. The role of inferior frontal areas in auditory working memory has also been implicated in several studies on working memory for pitch^41–44^. Thus, pitch processing could involve perceptual as well as cognitive processes. Our findings are consistent with these studies in terms of localizing active brain areas during pitch judgment tasks.

When comparing incongruent versus congruent conditions, differential activation was found in the inferior frontal areas. In a recent fMRI study, activation in the planum temporale posterior to the primary auditory area was reported during auditory scale illusion^45^. In the original Shepard tones and our modified Shepard tones, larger acoustic differences are inherent in the incongruent compared to the congruent conditions. When moving from one complex harmonic tone to another in an ascending sequence of Shepard tones, we shift pitch chroma one semitone up and pitch height one semitone up. However, when moving from the end to the beginning of a Shepard tone sequence, pitch chroma is shifted in one semitone while pitch height is shifted for almost an octave. This large acoustic difference could elicit bottom-up attention because of highly salient features in the stimuli. From our functional connectivity analysis, the left temporal areas showed an increased connectivity with bilateral frontal areas in the incongruent than congruent conditions. This result provides evidence for bottom-up information flow. Given the pitch judgment task used in our study, we speculate that such sensory information is first provided from the auditory cortex to higher-level processing areas through a bottom-up process. Then, this information is compared with prior knowledge via a top-down process involving cortical areas at higher processing levels. When the bottom-up information provides conflicting attributes, top-down processing is required to select features and resolve conflicting attributes based on prior knowledge. Thus, solving incongruent sensory cues of pitch could rely on interactions between the temporal and frontal cortices, or interactions between bottom-up and top-down processes.

Of particular interest is the stronger activation observed in the right inferior frontal areas during the incongruent conditions. The increased BOLD signals in the inferior frontal areas may reflect increased working memory in the pitch judgment task when directions of change in pitch height and pitch chroma cues were inconsistent. The right inferior frontal areas also exhibited an enhanced connectivity with their hemispheric counterparts in the left hemisphere in the incongruent compared to congruent conditions. Stronger connectivity within and across hemispheres in the frontal areas could be associated with additional processes required to resolve conflicting pitch cues. Specifically, when both the pitch height and pitch chroma information matches, only minimal cognitive processes are required. However, when the pitch height information contradicts the pitch chroma information, more demanding cognitive processes are required before a percept can be generated. Enhanced connectivity observed in the frontal areas can also be related to applying the Gestalt principles of proximity to resolve incongruent pitch cues and eventually achieve a stable percept of pitch shift. In the visual domain, inferior frontal activation was reported when visual perception involved the Gestalt principle of proximity^46^. Interestingly, resolving conflicting sensory cues in other domains also involved the inferior frontal areas. For example, inferior frontal areas were activated when solving conflicting sensory information for dot motion^23,47^ and conflicting task rules^48^. These findings taken together suggest the inferior frontal areas are potential sources of cognitive processes that require applying prior knowledge and resolving conflicting perceptual cues across domains.

One limitation of our current study is that perceptual salience was not equated between pitch height and chroma. This is because pitch chroma is categorical, but pitch height is not. However, our complex tones were discriminable in terms of pitch height and chroma. For pitch height, the smallest amount of pitch shift in our study was 5.95%, which was approximately 3-fold larger than the average f0 difference limen (DL) of 1.9% reported in Allen & Oxenham^6^. For pitch chroma, complex tones one semitone apart (e.g., A4, A#4) are readily discriminable by most listeners. Although both pitch height and chroma cues were discriminable in our study, we did not manipulate the perceptual salience of the pitch height and chroma cues. Future studies should equate the perceptual salience across these two dimensions to further address the reliance on pitch height and pitch chroma for processing complex harmonic tones.

In our current study, we varied both the harmonic partials and spectral centroid in the same direction. Perceptually, shifts in the lowest harmonic or fine structures are dominant cues for changes in pitch height, while shifts in the spectral centroid or envelope are primary cues for changes in timbre^6,49–51^. Allen & Oxenham^6^ reported interaction and interference effects between pitch height and timbre. Variations in timbre can interfere with pitch height perception^6,52^. By varying both pitch height and timbre in the same direction in our study, we reduced the interference of timbre on pitch height perception. However, because we changed both the pitch height and timbre cues in the same direction, listeners could use either the pitch height or timbre cue to perform the pitch judgment task. To direct subjects’ attention to pitch height, we instructed subjects to judge whether pitch was shifted downwards or upwards, instead of whether the timbre was duller or brighter. Although subjects’ attention was directed to pitch height through our instructions, subjects might still vary in using either the pitch height or timbre cue for pitch judgments. Further studies are required to explore the interaction among pitch height, timbre, and chroma for octave-spaced harmonic complexes.

In conclusion, by shifting harmonic partials and pitch chroma in an opposite direction, we created situations in which pitch height and pitch chroma provided conflicting cues of pitch shift. Behaviorally, we observed stable percepts of pitch based on the pitch chroma cue during the incongruent conditions. At the cortical level, the inferior frontal areas were found more active when comparing the incongruent and congruent conditions. Enhanced connectivity was observed from the left temporal to frontal areas and between hemispheric counterparts of the frontal areas. Our results suggest that resolving incongruent perceptual cues places higher demands on areas subserving top-down and bottom-up processes that solve conflicting pitch cues. To better understand the role of pitch chroma and pitch height in resolving conflicting pitch cues, further studies manipulating various acoustic parameters of complex harmonic tones are required. Future neuroimaging studies can also compare brain activation during pitch perception of incongruent and congruent cues in musical contexts to test the role of inferior frontal areas in solving incongruent pitch cues and forming stable percepts.

## Methods

### Subjects

Sixteen adult subjects with no reported formal musical training were recruited to this study (6 males and 10 females; mean age: 23.3 +/− 2.6 years, ranging from 21 to 29). All subjects provided written inform consent and the experimental protocols were approved by the Institute of Review Board at the National Taiwan University Hospital. All experimental methods were carried out in accordance with the approved guidelines. All subjects had no hearing or neurological problems.

### Stimuli

Complex tones of 350 ms duration were created and ramped at onset and offset for 10 ms. Each complex tone was comprised of seven harmonic partials an octave apart. Amplitudes of these harmonic partials were scaled by a Gaussian envelope. In Shepard’s original experiment, spectral envelopes remained the same for all complex tones while their harmonic partials shifted up or down. In our study, we varied both the center of the spectral envelopes and harmonic partials. Specifically, the spectral envelope/centroid and harmonic partials were shifted in the same direction. However, the direction of pitch shift in chroma was varied independently of the spectral envelope and harmonic partials (Fig. 1).

For simplicity, we referred to each complex harmonic tone in our study in terms of their central partial. For example, the complex tone A#5 had its spectral centroid at 932.33 Hz, corresponding to the frequency of the music note A#5. The seven harmonic partials of the complex tone A#5 ranged from A#2 (116.54 Hz) to A#8 (7458.62 Hz). The naming of complex tones here followed the music note naming convention with a letter indicating the equivalent note on the chromatic scale (or, pitch chroma), followed by a suffix of number indicating the octave number (or pitch height). Details of the frequency partials and their amplitudes were listed in Table 4.

**Table 4:** Complex harmonic tones used in the present study. Frequency partials (from the lowest to the highest; in Hz) and amplitudes (% relative to the maximum amplitude) are listed.

chroma	octave	f1	f2	f3	f4	f5	f6	f7	unit
B	**4**	61.74	123.47	246.94	493.88	987.77	1975.53	3951.07	Hz
A	**5**	110	220	440	880	1760	3520	7040	Hz
A#	**5**	116.54	233.08	466.16	932.33	1864.66	3729.31	7458.62	Hz
B	**5**	123.47	246.94	493.88	987.77	1975.53	3951.07	7902.13	Hz
A	**6**	220	440	880	1760	3520	7040	14080	
		10.54	36.79	77.88	100	77.88	36.79	10.54	%
		**f1**	**f2**	**f3**	**f4**	**f5**	**f6**	**f7**	
					(spectral centroid)				

Note: Each complex tone is listed by a letter representing the pitch chroma (e.g., A, A#, B), followed by a number indicating the octave in which the spectral centroid is placed (e.g., 4, 5, 6). For each complex tone, frequencies of their harmonic partials (from the 1^st^ harmonic **f1** to the 7^th^ harmonic **f7;** in Hz) and their amplitudes (% of the maximum sound levels) are listed. The center frequency partial, **f4**, has the highest amplitude.

Here, conditions were defined in terms of pitch change directions in pitch height (shifts in the harmonic partials) and pitch chroma (shifts in relative position within an octave as denoted by the music note, e.g., A or A#). Four conditions were tested. Figure 1 shows schematic illustrations of the four conditions and their complex tone pairs. These four conditions were ***(i) congruent condition up-up (UU)****:* a pair of complex tones with pitch height and pitch chroma shifted upwards, *e.g*., [A5, A#5]; ***(ii) congruent condition down-down (DD):*** a pair of complex tones with pitch height and pitch chroma shifted downwards, *e.g*., [B5, A#5]; **(iii)** ***incongruent condition down-up (DU)****:* a pair of complex tones with pitch height shifted downwards, but pitch chroma shifted upwards, *e.g*., [A6, A#5]; ***(iv) incongruent condition up-down (UD):*** a pair of complex tones with pitch height shifted upwards, but pitch chroma shifted downwards, *e.g*., [B4, A#5]. The congruent conditions (UU, DD) were designed to ensure that subjects could accurately perform pitch judgment tasks. For the incongruent conditions (DU, UD), the perceived pitch change could be upwards or downwards depending on which perceptual cue subjects assigned more weights to.

### Stimulus presentation

Complex sounds were presented in pairs. A 900-ms silent period was inserted between a pair of sounds. The second sound was followed by another silent period of 900 ms before a visual cue for motor response was presented. The visual cue was displayed on the screen with a random duration (between 1750 ms and 11750 ms) before the beginning of the next trial. This experimental design allowed temporal jitters between trials and sufficient time for subjects to respond before the beginning of the next trial. Stimulus presentation and timing were controlled by Presentation (version 16.5; Neurobehavioral Systems). The auditory stimuli were presented to subjects via MRI compatible insert earphones (Model S14, Sensimetrics). During fMRI scans, stimuli were presented at 83 dB SPL to ensure that sounds were both audible and discriminable for each subject.

### Experimental paradigm

Each subject was tested with one structural run and four functional runs. Each functional run lasted for seven minutes. Thus, the total measurement time in the scanner was about 35 minutes.

For each functional run, 14 trials of each condition were presented in random orders. With a total of 4 runs, 56 trials were tested for each condition in each subject. The same number of trials was tested across subjects. Subjects were instructed to decide whether the pitch went up or down for a given pair of complex tones. Subjects were required to press a button with their left thumbs when they perceived a downward pitch shift and with their right thumbs when they perceived an upward pitch change. During the baseline period, subjects maintained fixation at the fixation cross, which was displayed on the screen throughout each of the four functional runs. At the beginning of each experiment, subjects practiced outside the scanner to familiarize with the tasks.

### fMRI and MRI data acquisition

fMRI scans were performed on a 3 T MR scanner (MAGNETOM Skyra, Siemens, Erlangen, Germany). An echo-planar imaging (EPI) sequence was employed with repetition time (TR) = 2000 ms, echo time (TE) = 30 ms, flip angle = 90°, field of view (FOV) = 224 × 224 mm^2^, and slice thickness = 3.5 mm. Considering variability of timing in hemodynamic responses^53,54^ and limited numbers of volumes collected in sparse temporal sampling^55,56^, we used continuous sampling to acquire functional data. For each subject, structural images were also acquired using a three-dimensional *T*_1_-weighted pulse sequence (MP-RAGE sequence: TR = 2530 ms, TE = 3.30 ms, flip angle = 7°, FOV = 256 × 256 mm^2^, and thickness = 1 mm) in the middle of the session.

### fMRI data analysis

Preprocessing of EPI data was carried out with FSL 5.0.5 (FMRIB Software Library, www.fmrib.ox.ac.uk/fsl), including motion correction using MCFLIRT, slice timing correction, spatial smoothing using a kernel of FWHM 5 mm, and normalization. At the single-subject level, General Linear Model (GLM) was used to estimate hemodynamic responses, which were modeled by the convolution between onsets of the first stimulus in each sound pair and a double-gamma hemodynamic response function. In the first-level analysis, four basic contrasts were tested: (1) UU versus baseline, (2) DD versus baseline, (3) DU versus baseline, and (4) UD versus baseline. To find out cortical areas associated with resolving conflicting perceptual cues, one additional contrast was tested: incongruent [DU + UD] versus congruent [UU + DD]. For the higher-level group analysis, FLAME (FMRIB’s Linear Analysis of Mixed Effects) was used with automatic outlier detections^57–59^. Statistical results of the whole brain analysis were based on Gaussian random field theory to control for family-wise error (FWE)^60^. We used a cluster-extent approach with a height threshold of Z = 3.09 and a cluster threshold of *p* = 0.001 (corrected). Results were coregistered to the “fsaverage” template in FreeSurfer (http://surfer.nmr.mgh.harvard.edu) and shown on the cortical surface.

Conjunction analysis was performed using the FSLMATHS tool of FSL^61^ to identify areas commonly activated by the congruent and incongruent conditions. Four contrasts (e.g., [UU versus baseline], [DD versus baseline], [DU versus baseline], [UD versus baseline]) were combined and tested against the conjunction null hypothesis where one or more effects are null^61,62^. Conjunction analysis was performed with a height threshold of Z = 3.3 and a cluster threshold of *p* = 0.001 (corrected).

To compare functional connectivity between the incongruent and congruent conditions, we ran psychophysiological interaction (PPI) analysis^63–65^. PPI analysis identifies task-related changes in connectivity between a seed region and the rest of the brain. Seed regions were selected based on results obtained from the conjunction analysis, including the Heschl’s gyrus, planum polare, planum temporale, superior temporal gyrus (anterior), and inferior frontal gyrus (pars opercularis). To allow individual variability in the precise activation location, these seed regions were defined bilaterally based on the Harvard-Oxford cortical atlas available in FSL. For the first-level analysis, we used the mean time series data extracted from each seed region as the physiological regressor. The psychological regressor was a vector coding for the congruent and incongruent trials. The PPI regressor was the regressor of interest and represented the interaction between the physiological and psychological regressor. Group-level statistics were thresholded with a height threshold of Z = 2.3 and a cluster extent threshold of *p* = 0.05 (corrected).
